# Patterns of Benign and Malignant Lesions of the Thyroid in Two Wilayahs of Northeastern Algeria

**DOI:** 10.1155/2015/849416

**Published:** 2015-11-22

**Authors:** Mona Guidoum, Hind Kherfi-Kadi, Ouahiba Benharkat-Boughaba, Aicha Djemaa-Bendjazia, Sihem Keghouche, Behnoush Abedi-Ardekani, Amina Azzouz, Yacine Kadi, Pierre Hainaut, Zihad Bouslama

**Affiliations:** ^1^Research Laboratory “Ecology Terrestrial and Aquatic Systems” (EcoSTAq), Team “Emerging Diseases and Environment”, University Badji Mokhtar, Annaba, Algeria; ^2^Central Laboratory Pathological Anatomy, EPH, El-Taref, Algeria; ^3^Central Laboratory Pathological Anatomy, EPH, Ibn Zohr, Guelma, Algeria; ^4^Head Radiotherapy Oncology, Hospital Ibn Badis, University 3, Constantine, Algeria; ^5^Nuclear Medicine Department, Hospital Ibn Badis, University 3, Constantine, Algeria; ^6^International Agency for Research on Cancer, Lyon, France; ^7^Institut Albert Bonniot Inserm UJF 823, Grenoble, France

## Abstract

The aim of this study is to compare histological patterns and to estimate the burden of thyroid cancers in the two Wilayahs (departments) of El-Taref and Guelma in northeast of Algeria (total population 0.9 million), locally reputed as having different rates of endemic thyroid diseases and cancer. A retrospective analysis of central pathology registers and clinical records of patients with thyroid diseases, covering the period 2008–2012, was conducted. A total of 145 cases of thyroid cancers with histological confirmation were registered in the two Wilayahs during the period, with a female to male ratio of 5.9 : 1. Estimates of crude incidence rates suggested that thyroid cancers were twice as frequent in the Wilayah of Guelma compared to El-Taref (*p* < 0.05) with a tendency to occur at a younger age in resident of the Wilayah of El-Taref. Diagnoses of thyroid adenoma were more frequent in the Wilayah of Guelma, whereas the prevalence of other thyroid lesions, including goitre, was similar in the two Wilayahs. This first descriptive study on geographic variations in thyroid cancer in Northern Africa suggests that significant differences may occur in relation with environmental and lifestyle exposures.

## 1. Introduction

Worldwide, thyroid cancer (TC) accounts for about 1–5% of all cancers. Incidence rates are consistently higher by 3–7-fold in females than in males and range from less than 1 per 10^5^ person-years (in sub-Saharan African males) to over 10 per 10^5^ person-years (in Caucasian North American females) [1].

TC is classified in distinct histological subtypes, including papillary (PTC), follicular (FTC), modularly (MTC), poorly differentiated (PDTC), and anaplastic (ATC) carcinomas [2]. In recent years, a sharp and significant increase in rates of TC has been observed in most parts of the world, in particular in western countries and in Asia, where in some places incidence rates have been near-doubled over the past decade [3]. There is debate as whether this increase reflects greater awareness and more extensive diagnosis or profound changes in the distribution of risk factors that may play a role in TC [3, 4]. Risk factors for TC include exposure to radiation, diabetes and obesity, tobacco smoking, and chronic diseases of the thyroid caused by iodine deficiency, autoimmune conditions (Grave's disease), or inflammatory diseases [4–7]. About 25% of MTC occur in a familial setting in subjects with mutations in the* RET* gene that predisposes to Multiple Endocrine Neoplasia Type IIA (MENIIa) [5, 8]. Furthermore, a recent study of the risk of TC in a population-based cohort in five Nordic European countries has identified a threefold increase of the risk in first-degree relatives of patients with primary TC [9].

In Africa, relatively stable rates of TC have been observed in recent years, and these rates are lower than in most other parts of the world [10, 11]. However, the disease is largely underdiagnosed and underreported, and information on the patterns of chronic thyroid conditions and histological subtypes of TC is scarce [3, 10]. The highest estimated incidence rates are reported for northern African countries. In Algeria, estimated crude incidence rates are of 1.2 and 7.1/10^5^ person-years in males and females, respectively [12].

In this study, we have compared the patterns of chronic diseases of the thyroid and of TC in two Wilayahs (districts) of northeastern Algeria, El-Taref and Guelma, which are locally reputed as having different rates of endemic thyroid diseases and TC. These two Wilayahs are not covered by population-based cancer registration. To estimate the burden of thyroid diseases, we have taken advantage of the existence of a single, centralized system of pathology referral in each of these districts to conduct a retrospective analysis and evaluation of pathological archives for the period of 2008–2012. These data were complemented by information extracted from the clinical records from the Nuclear Medicine Department of the University Hospital Ibn Badis in Constantine, a major referral center for patients residing in the Wilayah of Guelma. Our results show that the patterns of chronic diseases of the thyroid as well as of histological subtypes of TC are different in the two Wilayahs, with the highest estimated rates in the Wilayah of Guelma, an area of endemic disease of the thyroid.

## 2. Methods

### 2.1. Populations and Data from Pathology Registers

**Figure 1:**
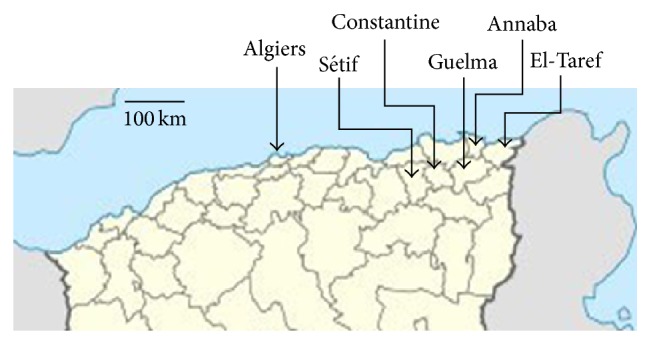
Localisation of data sources. The original data on thyroid lesions used in the study were extracted from the registries of the Departments of Pathology of District Hospitals in Guelma and El-Taref (Eastern Algeria), as well as from clinical records from a tertiary referral treatment center in the nearby city of Constantine. Data on cancer incidence from two population-based cancer registries in Algiers and Sétif were used for comparison.

This study is a retrospective study of hospital archives on period of 5 years from 1 January 2008 to 30 December 2012. The primary data were extracted from the archives of the Central Laboratories of Pathology of two main public referral hospitals, the El-Taref Hospital (in the Daira of El-Taref) and the Ibn Zohr Hospital (in the Daira of Guelma). These laboratories serve as main reference centers for pathology in their respective geographical administrative Wilayahs (districts), located in the northeastern part of Algeria (see map on Figure 1). Patients with clinically or cytologically suspicious lesions were treated by partial or complete surgery and histologically confirmed lesions of the thyroid were included in the study. Histological diagnosis was independently confirmed by two pathologists in El-Taref and by three pathologists in Guelma. Information on age, gender, and place of residence was anonymously extracted. In addition, data were extracted from the archives of the Department of Nuclear Medicine of University Hospital Ibn-Badis, Constantine, the main tertiary referral center for treatment of TC in northeastern Algeria. Information on patients residing in the Wilayahs of Guelma and El-Taref was extracted, crossed with information of the Central Laboratories of Pathology of the two districts, and duplicates were eliminated.

### 2.2. Data from Cancer Registries

Data on crude incidence rates of TC in three population-based cancer registries in Algeria were compiled over the period 1993–2010. For the cancer registry of Algiers, data were obtained from cancer incidence in five continents (CIV) Vol. 8 (1993–1997) [15] and from cancer registry reports for years 2003, 2006, and 2007 [16–18]. Time trends of cancer incidence in Setif, Algeria, 1986–2010 were recently reported [19] and additional data were obtained from CIV Vol. 6 (1986–1989) [13], Vol. 7 (1990–1993) [14], and Vol. 9 (1998–2002) [20] and Vol. 10 [21] whereas data for years 2006 and 2010 were extracted from cancer registry reports. For the registry of Annaba (not included in CIV), unpublished registry reports for the period 2003–2009 were used.

### 2.3. Estimates of Crude Incidence Rates and Statistical Analysis

Annual populations for years 2008 to 2012 for districts of Guelma and El-Taref (5-year age groups) were obtained from the National Office of Statistics, Algeria (http://www.ons.dz/IMG/pdf/pop3_national.pdf). The population of year 2010 (mid-point population) was used as basis to estimate crude incidence rates in these districts. Ten-year age groups were compiled by aggregating population data for 5-year age groups. Comparison between numbers and distribution of cases between the two Wilayahs were performed using standard Chi-squared test, with tools available at http://vassarstats.net/.

## 3. Results

Figure 1 shows the localization of the data sources used for the present study. Two types of data were used, including cancer registry data from Algiers, Setif, and Annaba, covering an essentially urban population, and data extracted from pathology archives and hospital clinical files in the Wilayahs of Guelma and of El-Taref, northeastern Algeria. These two Wilayahs differ by their population and ecological context. Whereas the coastal Wilayah of El-Taref (population in 2010: 408,414) is mostly urbanized (7 Dairas (subprefectures) and 24 municipalities) (http://www.andi.dz/PDF/monographies/Tarf.pdf), the semimountainous Wilayah of Guelma (population in 2010: 482,430) is largely rural (10 Dairas (subprefectures) and 34 municipalities) (http://www.andi.dz/PDF/monographies/Guelma.pdf) and is considered, based on local doctor's experience, as a region of endemicity for goiter.  Table 1 compiles the numbers of patients with histopathologically defined lesions of the thyroid diagnosed in the Wilayahs of Guelma and of El-Taref for the period 2008–2012. Overall, a total number of 256 cases were diagnosed in Guelma, compared with 191 cases in El-Taref. In both Wilayahs, most patients were females (M/F ratio of 1 : 6.1 in Guelma and of 1 : 12.7 in El-Taref). Strikingly, the proportion of thyroid lesions diagnosed as “neoplastic” was much higher in Guelma (65.2%) than in El-Taref (15.7%). Among 131 cases of TC, 99 (75.6%) were PTC, 22 (16.8%) were FTC, 7 (5.3%) were hyalinizing trabecular tumors, 2 (1.5%) were MTC, and 1 (0.8%) was ATC. The mean age at diagnosis of TC was 45.4 ± 16.3 years in males and 42.6 ± 14.6 years in females. Follicular adenoma was also more represented in Guelma (*n* = 58, 22.7% of all lesions) than in El-Taref (*n* = 8, 4.2%). Most of nonneoplastic lesions were hyperplasia.

**Figure 2:**
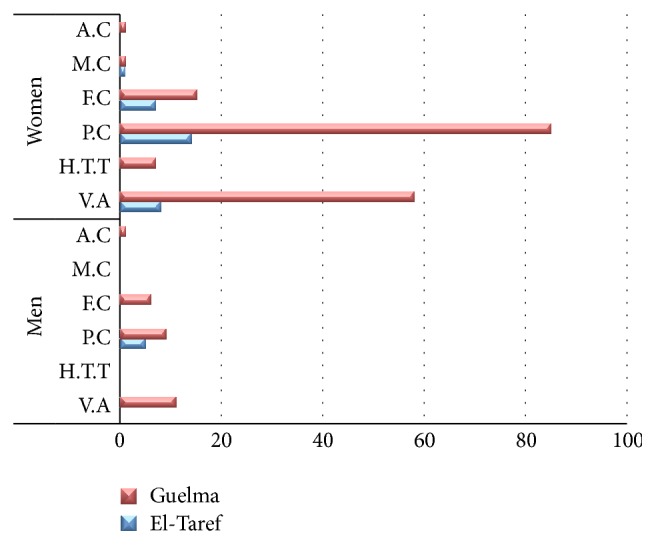
Distribution of different forms of neoplastic lesions of the thyroid in men and women in the districts of Guelma and of El-Taref. A.C: anaplastic carcinoma, M.C: medullary carcinoma, F.C: follicular carcinoma, P.C: papillary carcinoma, H.T.T: hyalinizing trabecular tumors, V.A: follicular adenoma.

Figure 2 shows the distribution of different types of lesions of the thyroid according to 10-year age groups among male and female patients of the two Wilayahs. For each category of lesions including TC, the peak 10-year age group was 26–35 years in the Wilayah of El-Taref, compared to 36–45 years in the Wilayah of Guelma, suggesting that lesions tended to be diagnosed at an earlier age in the Wilayah of El-Taref.

Table 2 shows estimates of crude incidence rates for TC in the two Wilayahs and compares them with available data on crude incidence of TC in cancer registries from Algeria. The estimated crude rates for males were 3.3 in Guelma and 1.2 in El-Taref. For females, they were 21.14 in Guelma and 5.39 in El-Taref. For the Wilayah of El-Taref, these figures are in the same range as crude rates of TC published in recent reports of population-based cancer registries from Algiers, Setif, and Annaba, while crude rates of the Wilayah of Guelma are significantly higher, representing to our knowledge the highest rates reported so far on the African continent.

## 4. Discussion

Thyroid cancer (TC) accounts for approximately 2% of all cancers diagnosed worldwide and 95% of all endocrine cancers [22]. Recent reports describe a continuous increase in TC incidence worldwide. In certain geographical areas, this increase exceeds 100% (and is as high as 250% in some places) [1]. By contrast, small declines in incidence were registered in a few areas.

In this study, we have used hospital-based tissue archives and patient files of the pathology departments of two central hospitals in the Wilayahs of Guelma and El-Taref, Algeria, to evaluate the numbers, histological definition, and age distribution of cancers of the thyroid in two areas of different population and ecological contexts. These administrative divisions encompass a population of about 0.9 million, which is not covered by population-based cancer registration. El-Taref is a coastal, densely populated, and mostly urbanized area whereas Guelma is a rural and semi-mountainous area. In each district, the central hospitals are the main primary and secondary referral site for the vast majority of the population. Thus, they represent, if not the exclusive, the majority source of diagnosis for lesions of the thyroid in these areas, enabling an estimate of crude incidence rates in the population of these two Wilayahs. Results show a striking difference in the distribution of neoplastic versus nonneoplastic lesions of the thyroid in the two Wilayahs. Whereas neoplastic lesions represented 65.23% of all diagnoses in the Wilayah of Guelma, they represented only 15.71% of the cases in the Wilayah of El-Taref. In both Wilayahs, PTC represented the main form of TC, followed by FTC, whereas MTC and ATC were infrequent. Comparison between age groups in the two Wilayahs suggests that patients with TC tended to be diagnosed at an earlier age in El-Taref as compared to Guelma. This difference may be due to better awareness of signs and symptoms of thyroid disease and earlier referral in the Wilayah of El-Taref. In the latter Wilayah, the estimates of crude incidence of TC (using the population of year 2010 as denominator) were in the same range as in recent reports (after 2006) of cancer registries in Algeria. In contrast, the crude incidence rates estimated for the Wilayah of Guelma were at least the double of the highest rates recently reported in cancer registries of Algeria and are the highest ever reported on the African continent. These estimates of crude rates of TC (as well as recent data from Algerian cancer registries) are much higher than reported in the current literature, which is mostly based on numbers reported over 10 years ago [1]. This observation suggests that, following a similar trend as in many industrialized countries, the rates of TC tend to increase in Algeria, probably as a result of improved detection and diagnosis. Furthermore, our results suggest that there may be important geographic disparities in the frequency and age of occurrence of TC. These results are compatible with a recent analysis of time trends in cancer incidence in the population-based cancer registry of Setif during the period 1986–2010 [21]. During that period, the incidence of TC (age-standardized rate, world standard) grew from 0.3 to 1.4 persons/10^5^ years in males and from 0.9 to 6.0 persons/10^5^ years in females, representing an annual percent change (APC) of +3.2% (95% CI (−3.6; +10.5)) in males and +5.3% (95% CI (+2.8; +7.9)).

Although our study does not directly address the nature of the factors causing these disparities, environmental risk factors such as iodine deficiency have been commonly suspected as a possible cause. The area of Guelma is known by local doctors as an area of endemicity for goiter, although precise documentation of this information is not available. Of note, Algeria is implementing a nationwide policy of salt iodine supplementation since the early 1990s. However, data on precise levels of iodine in different populations are not available. On the other hand the differences observed here might be the consequence of differences in access to diagnosis and care, with patients in Guelma tending to access diagnosis later than those of El-Taref, thus presenting with a higher proportion of neoplastic lesions and at later age. Such a discrepancy in access to diagnosis and care may be caused by multiple factors, including lack of awareness of patients in different socioeconomic and development context (more traditional/rural in Guelma, more urban and industrialized in El-Taref). Therefore, further approaches aimed at improving information and early detection of thyroid lesions may be warranted in rural areas.

## 5. Conclusion

These results show contrasting rates of incidence of TC in two Wilayahs of northeastern Algeria (El-Taref and Guelma). The very high rates observed in Guelma strongly suggest that strong environmental risk factors have an impact on this population. Our study is limited by the fact that it is not based on formal, population-based cancer registration. On the other hand, its strength is that it uses high-quality, centralized pathology reports, providing an accurate basis for diagnosis. Further studies are needed to determine whether preventive intervention may help to curb the high rates of TC observed in Guelma. Furthermore, studies on the incidence of TC in other parts of rural Algeria are warranted.

## Figures and Tables

**Table 1:** Histopathological definition of cases.

	District of Guelma					District of El-Taref				
	Men		Women		M/F ratio	Men		Women		M/F ratio
	Number	%	Number	%		Number	%	Number	%	
All lesions	42	8.33	256	50.79	1 : 6.1	15	2.98	191	37.9	1 : 12.7
*Nonneoplastic*										
Hyperplasia	11	2.18	74	14.68	1 : 6.7	10	1.98	149	29.56	1 : 14.9
Thyroiditis										
Hashimoto's	0	0.00	2	0.4	—	0	0.00	4	0.79	—
Lymphocytic	0	0.00	11	2.18	—	0	0.00	2	0.4	—
Other	4	0.79	2	0.4	1 : 0.5	0	0.00	6	1.19	—
*Neoplastic*										
Follicular adenoma	11	2.18	58	11.51	1 : 5.3	0	0.00	8	1.59	—
Hyalinizing trabecular tumors	0	0.00	7	1.39	—	0	0.00	0	0.00	—
Papillary carcinoma	9	1.78	85	16.86	1 : 9.4	5	0.99	14	2.78	1 : 2.8
Follicular carcinoma	6	1.19	15	2.98	1 : 2.5	0	0.00	7	1.39	—
Medullary carcinoma	0	0.00	1	0.20	—	0	0.00	1	0.2	—
Anaplastic carcinoma	1	0.20	1	0.20	1 : 1	0	0.00	0	0.00	—

**Table 2:** Estimates of crude incidence rates of cancers of the thyroid in different Wilayahs of Algeria.

Wilayahs^(a)^	Period	Crude rates, per 100,000 person-years		Data source
		Men	Women	
Guelma	2008–2012	3.31	21.14	Hospital-based data, this study

El-Taref	2008–2012	1.2	5.39	Hospital-based data, this study

Algiers	1993–1997	0.9	3.5	CIV-8^(b)^
	2003	1.7	7.7	Population-based cancer registry^(c)^
	2006	1.2	8	
	2007	1.8	6.3	

Setif	1986–1989	0.2	0.8	CIV-6 [13]
	1990–1993	0.1	0.4	CIV-7 [14]
	1998–2002	0.9	2.6	CIV-9^(d)^
	2006	0.8	4.0	Population-based cancer registry^(d)^
	2010	1.5	6.0	Population-based cancer registry^(d)^

Annaba	2003–2009	1	3.1	Population-based cancer registry^(e)^

(a): see Figure 1 for localization of data sources.

(b): cancer incidence in five Continents, IARC, (http://ci5.iarc.fr/) [15].

(c): Registres des Tumeurs d'Algers, 2003, 2006, and 2007 [16–18].

(d): cancer registry of Setif; for age-standardized incidence rates (world standard) see [19].

(e): Rapport du Registre des Cancers d'Annaba, 2003–2009, unpublished (personal communication, Dr Bouzbid).
